# Characterization and Biological Activity of Fiber-Type *Cannabis sativa* L. Aerial Parts at Different Growth Stages

**DOI:** 10.3390/plants11030419

**Published:** 2022-02-03

**Authors:** Giulia Mastellone, Arianna Marengo, Barbara Sgorbini, Federica Scaglia, Francesca Capetti, Francesco Gai, Pier Giorgio Peiretti, Patrizia Rubiolo, Cecilia Cagliero

**Affiliations:** 1 Department of Drug Science and Technology, University of Turin, 10125 Turin, Italy; giulia.mastellone@unito.it (G.M.); arianna.marengo@unito.it (A.M.); barbara.sgorbini@unito.it (B.S.); federica.scaglia@edu.unito.it (F.S.); francesca.capetti@unito.it (F.C.); patrizia.rubiolo@unito.it (P.R.); 2 Institute of Sciences of Food Production, National Research Council, 10095 Grugliasco, Italy; francesco.gai@ispa.cnr.it (F.G.); piergiorgio.peiretti@ispa.cnr.it (P.G.P.)

**Keywords:** *Cannabis sativa*, phytochemical fingerprint, growth stage, method of drying, non-psychotomimetic cannabinoids, flavonoids, antioxidant assays, tyrosinase inhibition, UHPLC-UV-ESI-MS/MS

## Abstract

Currently, there is a renewed interest in cannabis-related products in different fields because of the rich phytocomplex of this plant, together with its fiber and agricultural features. In this context, the current study aims to chemically characterize different samples of fiber-type *Cannabis sativa* L. grown in Italy as a potential health promoting source. An ultrasound-assisted solid-liquid extraction (UA-SLE) method was first developed and optimized to obtain a fingerprinting of the investigated phytocomplex. Analyses were carried out through an ultra high performance liquid chromatography equipped with a photodiode array detector in series with triple quadrupole system with an electrospray ionization (ESI) interface (UHPLC-UV-ESI-MS/MS) and showed that the phytocomplex mainly includes flavonoids and non-psychotomimetic cannabinoids. The method was then applied to characterize and compare 24 samples of fiber-type *Cannabis sativa* L. aerial parts (mainly stems and leaves), which differed for the growth stages (from mid-vegetative to early flowering), growth land plots, and methods of drying (forced-draft oven or freeze-drying). The quali-quantitative analysis showed that a freeze-drying method seems to better preserve the chemical composition of the samples, while the location of the land plot and the growth stage of the plant (which did not comprise inflorescences) had minor influences on the chemical pattern. These results were also supported by spectrophotometric in-vitro assays (scavenging of 2,2-diphenyl-1-picrylhydrazyl (DPPH^•^) and 2,2′-azinobis-3-ethyl-benzthiazoline-6-sulphonate (ABTS^+•^) radicals and inhibitory activity against tyrosinase and elastase enzymes) to investigate the potential biological activity of these samples and the contribution of non-psychotomimetic cannabinoids.

## 1. Introduction

The valorization of plants as natural and eco-compatible resources and as reservoirs of bioactive compounds has become increasingly important for several applications (e.g., textiles, cosmetics, nutraceuticals, and pharmaceuticals), in the perspective of sustainable growth and development [1,2]. *Cannabis sativa* L. (family: Cannabaceae) perfectly fits this purpose because of its versatility and potentiality. It is a dioecious (rarely monoecious) herbaceous annual plant, native of Central Asia but easily adaptable to different geographical conditions. Known since ancient times for its textile, fiber, and chemical features, in the 1990s its cultivation was characterized by a slow decline because of the psychotropic effects of one of its major compounds, delta^9^-tetrahydrocannabinol (Δ^9^-THC) [3,4]. Currently, a renewed interest for this species is opening the way for its exploitation in different fields.

There is no unanimous agreement about its taxonomy, but the most common refers to a single species (*C. sativa*) with different chemotypes based on the “total cannabidiol (CBD)”/“total THC” ratio. The drug type, richer in Δ^9^-THC, and the fiber type, richer in CBD or related compounds (commonly known as hemp or industrial hemp), are the most important from the economic point of view. The first chemotype is normally subjected to restrictions according to the legislations of individual countries, while the second is the main source for the cannabis market, which includes food and personal care products but also medical formulations [5,6,7]. Despite the attention that has been mostly focused on Δ^9^-THC and CBD, the chemistry of hemp is very complex. Numerous chemicals are synthetized in the different plant parts (inflorescences, leaves, stems, and seeds), including terpenes, carbohydrates, fatty acids, and their esters, amides, amines, phytosterols, phenolic compounds, and non-psychotomimetic cannabinoids that have been associated with health-promoting properties, e.g., neuroprotective, antioxidant, and anti-inflammatory effects [4,5,6,8,9,10].

The stem is one of the main agricultural products of the fiber chemotype because of its cellulose and woody fibers that are used as animal feed, but also to produce bioplastics and, of course, in the textile and paper sectors. Seeds are also considered important raw materials for their unsaturated fatty acids content and for the production of hemp oil, used as a valuable ingredient in cosmetic and nutraceutical preparations. Leaves and inflorescences are normally considered waste in the fiber industry, but their valorisation is increasing because of the above-mentioned chemical characteristics [3]. Although clear regulations on cannabis-derived products and by-products in the Italian and European market are lacking, a systematic characterization of the chemical profile and biological activity of these parts of the plant is needed. In fact, currently it is possible to find different *C. sativa*-based products on the market in form of food supplements, cosmetics, resin, oil, and e-liquid for electronic cigarettes [11]. While most studies have been directed toward the analysis of hemp inflorescences and their biomarker compounds, the phytocannabinoids [6,12,13,14,15,16], little information is available on leaves and stalks [9,17,18] and on other specialized metabolites, such as polyphenols and derivatives that are characterized by several biomedical and pharmacological properties [4]. In fact, apart from hemp seeds and derivatives, leaves and sprouts of this plant are also authorized to be ingredients for cosmetic preparations, according to the CosIng European database (data updated to 9 November 2021). Besides the considered plant part, the age of the plants, the growth conditions, harvest times, and storage of the hemp can significantly affect the chemical composition and, more importantly, the cannabinoids content [19]. Much is known about the variations that occurs in the chemical pattern of cannabis, before and after flowering [20]. However, Westerhuis et al. [21] suggested that hemp for textiles should be harvested before flowering; therefore, a careful investigation of the previous growth stages is also needed.

Taking into account the above considerations, this study is focused on the characterization of 24 samples of fiber-type *Cannabis sativa* L. aerial parts, grown in the Western Po Valley (NW Italy) and collected during four progressive growth stages, before flowering. An ultrasound-assisted solid-liquid extraction (UA-SLE) method, coupled with UHPLC-UV-ESI-MS/MS analysis, was first optimized through an experimental design approach in order to obtain a fingerprinting of the investigated phytocomplex. Also, the influence of sample treatment was considered, as the plant was subjected to oven- or freeze-drying after harvesting. Finally, different colorimetric in vitro assays (scavenging of the 2,2-diphenyl-1-picrylhydrazyl (DPPH^•^), the 2,2′-azinobis-3-ethyl-benzthiazoline-6-sulphonate (ABTS^+•^) radicals, and inhibitory activity against tyrosinase and elastase enzymes) were carried out for preliminary considerations of the biological activity of the cannabis leaves and stem-based extracts that may be exploited in the cosmetics field. Considering the well-known antioxidant activity of flavonoids, both methanolic (richer in flavonoids) and acetone extracts (richer in cannabinoids) were tested to investigate the potential contribution of cannabinoids to the total antioxidant power of the samples. Tyrosinase and elastase are among the enzymes involved in skin aging and several natural inhibitors have already been proposed as ingredients for anti-aging products [22]. The anti-tyrosinase and elastase activity of these extracts was therefore also evaluated through an in vitro inhibitory test.

## 2. Results and Discussion

### 2.1. Optimization of the UA-SLE Method 

**Figure 1:**
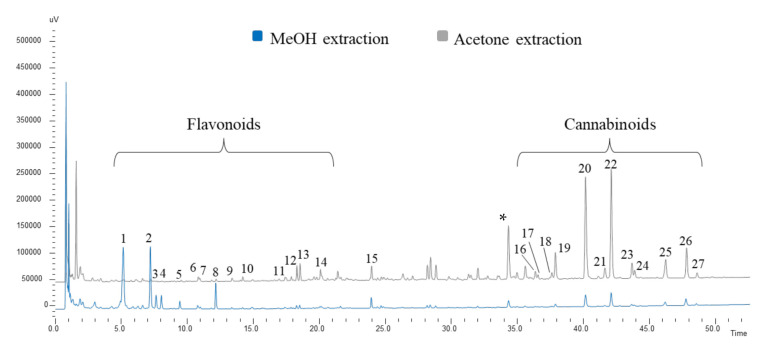
Chromatographic profile at 254 nm of methanolic and acetone extraction of fiber-type *Cannabis sativa* L. aerial parts at 5 mg/mL. For peaks identification, see Section 2.2. *: contaminant.

The optimization of sample treatment before chromatographic downstream analysis is a fundamental step to achieve better extraction efficiency. This is particularly true for complex matrices, such as plant samples, which normally need several pre-treatments before injection, especially when a liquid chromatography (LC) system is employed. In this case, different variables were taken into account for the extraction of fiber-type *Cannabis sativa* samples. To simplify the optimization study, a representative mix of all 24 samples was prepared, taking an equal amount from each sample. According to previous works that deal with plant analysis [23,24,25,26], an ultrasound-assisted solid-liquid extraction (UA-SLE) method was selected, it being efficient and easy to perform. Two solvents were tested for the solid-liquid extraction: methanol (MeOH), which presents a good affinity for different classes of compounds, while acetone was reported to have a good solubility for cannabinoids [27,28]. This was confirmed by our results, where the methanolic extraction allowed the extraction of a higher number of compounds belonging mainly to the classes of flavonoids and non-psychotomimetic cannabinoids, while the acetone extraction was more effective in selectively isolating cannabinoids (see Figure 1). Some works report the use of ethanol for the selective extraction of cannabinoids; however, our results showed that the ethanol extraction capacity was similar to that exerted by MeOH and not superior to that of acetone (Figure S1). The higher affinity of cannabinoids to acetone was probably caused by their lower polarity compared to flavonoids; for this reason, in the following experimental design the addition of water (more polar) was not considered in the subsequent optimization experiments [29].

An experimental design was employed for the optimization of the other variables considered, which include the sample amount (mg), the volume of solvent (mL), the percentage of water in the solvent (%) (considered only for MeOH extraction), and the time of extraction (min) with ultrasound (US). A full two-level factorial design (2*^n^*, with *n* = 4/3 variables) was used for the screening study, which comprises 16 (MeOH) or eight (acetone) experiments combining the minimum and maximum values considered for each factor. Moreover, three replicates of the central point (intermediate value for each variable) were carried out to monitor the reproducibility of the method. For the sample amount, the low and high limits were set at 100 and 500 mg, the volume of solvent ranged between 5 and 50 mL, while the time of extraction with US was evaluated between 10 and 30 min. Regarding the percentage of water, a maximum value of 50% was set for the extraction in MeOH to evaluate the influence of water in the extraction of the target analytes. For all the factors under study, the values were set with the aim of reducing the time of analysis and the consumption of materials (see Table S1). Once the experiments were performed, the extraction efficiency was evaluated in terms of the peak areas obtained for all the target compounds. The results are shown in the Pareto charts, Figure S2. For both the solvents, the increasing of the sample amount led to a higher extraction efficiency. However, the ratio between the peak area and the amount of the sample showed a linear trend without an exponential increasing of the performance of extraction. Considering the good sensibility of the extraction method, already obtained with 100 mg of the sample, the employed amount was set to the low value—also to reduce the consumption of raw material. Regarding the methanolic extraction, the other variable that truly affects the peak area was the content of water. While the amount of water was not significant for the flavonoid compounds, the extraction of cannabinoids was negatively influenced by a higher amount of this polar solvent and, therefore, the percentage of water was set at its lower value (zero). For the extraction in acetone, the most influential variable was the time of extraction, which also has a negative effect on the extraction efficiency of the cannabinoids. This may be caused by the slight increasing of temperature during the longer extraction times with US, as cannabinoid acids are sensitive to high temperatures [30]. Based on these results, for both the solvents the minimum value for all factors was selected as the optimum condition. Moreover, the elimination of water from the solvent of extraction allowed a reduction of the time of solvent evaporation needed to obtain the dried extract (see Section 3.4). Therefore, the optimized method required 100 mg of sample, 5 mL of solvent, and 10 min of extraction with US. Once the method was optimized, the exhaustivity of the extraction was evaluated for both solvents, considering the decrease of peak area for each compound, after five cycles of extraction on the same matrix. Three consecutive extractions allowed the recovery of 88% and 96% of the total amount of the components for MeOH and acetone, respectively, and were therefore selected for subsequent analyses. The optimized method was then applied for the extraction of the 24 samples of *Cannabis sativa* L.

### 2.2. Fingerprinting of a Representative Cannabis sativa Sample and Identification of the Target Compounds

A fundamental step in this study was the qualitative characterization of the plant sample. According to the existing literature [6,12,13,14,15,31,32], most of the studies on *Cannabis sativa* L. focused on the phytocomplex of the inflorescences and on cannabinoids that are synthetized in large amount in these plant parts. Our samples were collected at different growth stages (before flowering) and mainly consisted of stems and leaves. The same mix of all the samples used for the optimization (Section 2.1) was employed to investigate the fingerprinting of the plant. The extractions, following the optimized method, were performed both in MeOH and acetone and followed by HPLC-UV-ESI-MS/MS untargeted metabolite analysis. The chromatographic profile obtained at 254 nm is illustrated in Figure 1. The UV spectra for each peak provided a preliminary indication of the classes of compounds. The molecular weight was also defined by mass spectrometric data in SCAN mode through the complementary correspondence between positive and negative pseudomolecular ions in the ESI^+^ and ESI^-^ modes. The product ion scan analysis of the pseudomolecular ions under investigation provided diagnostic fragments for each compound. Based on UV and MS data, 27 informative peaks were identified or tentatively identified and selected as target compounds for the statistical analysis. All the information about the analytes under study are reported in Table 1.

Two main groups of compounds were identified: the more polar flavonoids and non-psychotomimetic cannabinoids, which are reported to be among the main class of phytochemicals produced by hemp [4,10,35]. The flavonoids were mainly in the form of glycosides and the most abundant (confirmed by co-injection with authentic commercial standards) were luteolin and apigenin as *O*-glucuronide derivatives. Other flavonoid glycosides detected in lower amounts, including acacetin and diosmetin derivatives, were tentatively identified through their tandem mass spectrometry fragmentation pattern. The MS/MS fragmentation gave a potential aglycon of 300 g/mol for peaks 3, 4, which corresponds to diosmetin molecular weight, while for peaks 5, 6, 7, and 8 the fragment was 284 g/mol, corresponding to acacetin. While apigenin and luteolin are normally reported in *Cannabis sativa* phytocomplex [4,9], to the best of the authors’ knowledge no data are available on the presence of acacetin and diosmetin glycosides in hemp samples. However, their corresponding aglycones were found in traces in the samples and confirmed with reference standards, supporting the identification data. Interestingly, Pollastro et al. [4] reported two flavones (canniflavone 1 and 2), isolated in 1980 and only found in cannabis, with close structural similarity to diosmetin.

In accordance with the phenotype of the plant (fiber-type), only non-psychotomimetic cannabinoids were found. In this case, few of these terpenophenolic compounds are easily available as commercial standards [19] and only cannabidiolic acid (CBDA), cannflavin A, and cannabichromenic acid (CBCA) were confirmed. The other analytes, which presented similar retentions times and maximum UV adsorption typical of cannabinoids in the acid form (around 220, 270 and 300 nm, see Table 1), were tentatively identified through their tandem mass spectrometry fragmentation patterns, which were compared with literature data [5,12,33]. As an example, the main cannabinoids (peaks 20, 21, and 22) in the extracts were putatively identified as varinic acid derivatives (propyl cannabinoids), which presented pseudomolecular ions 331 m/z and 329 m/z, in ESI^+^ and ESI^−^ ionization modes, respectively and were fragmented to give diagnostic ions at m/z 313 (water loss), 257, 233, and 191 in ESI+ (see Figure S3). Among the main propyl cannabinoids, tetrahydrocannabivarinic acid (Δ^9^-THCVA) and cannabivarinic acid (CBDVA) had similar fragments losses with some differences in ion intensity that are in agreement with our results [33]. A phthalate derivative (*) was also identified as a contaminant in the hemp extracts. Its abundance was higher in the oven-dried samples where the higher temperature may had caused a major release from an unknown source of contamination [36].

To support these results, a GC-MS analysis of the acetone extract, which presented a higher content of cannabinoids, was carried out. In particular, the extract was derivatized with *N,O*-Bis(trimethylsilyl)trifluoroacetamide (BSTFA) before the injection to avoid the decarboxylation of the cannabinoid acid, which easily forms the neutral species in the presence of high temperatures [30,35]. The GC analysis confirmed the presence of propyl cannabinoids CBDA, CBCA, and Δ^9^-THCA from the comparison with commercially available mass spectral libraries and the injection of CBDA and CBCA authentic commercial standards (see Figure S4 for GC-MS profile and spectra). 

As mentioned before, leaves and stems (which are the main components of the sample under investigation) had a lower content in cannabinoids [9,11] compared to inflorescences and few data were available for comparison purposes. Besides CBDA, Δ^9^-THCA, CBNA, CBGA, and CBCA were the other predominant chemicals of the class, depending also on the chemotype [10,35]. 

### 2.3. Analysis and Quantification of the Phenolic Compounds and Cannabinoids in the Samples

**Figure 2:**
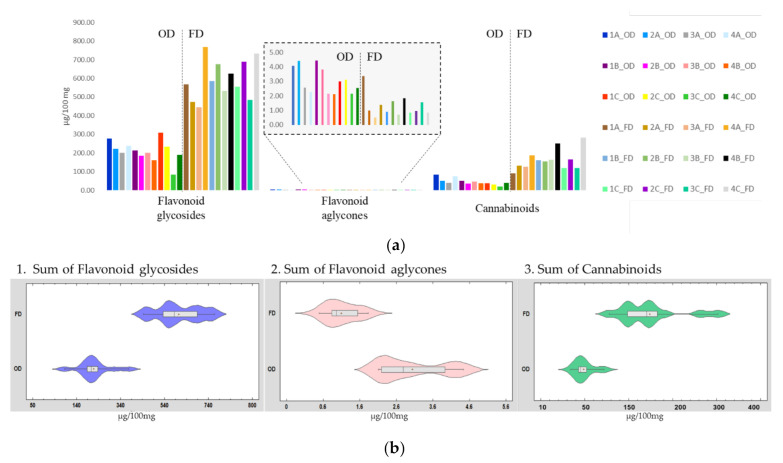
Representation of the quantification data of hemp methanolic extracts, expressed as µg of compound in 100 mg of sample: (**a**) distribution of the target analytes in the samples (sample code: 1_4 growth stage; A_C land plot); and (**b**) violin plot of quantification results for flavonoid glycosides, aglycones and cannabinoids in the freeze-drying (FD) and oven-drying (OD) samples.

The most abundant phenolics and cannabinoids were quantified by LC-PDA analysis using the external calibration method (Table S2). The quantification of the 24 samples was performed on the methanolic extracts that had a more complete phytochemical profile. When available, the corresponding authentic commercial standard was used to build the calibration curve (luteolin-7-*O*-glucuronide, apigenin-7-*O*-glucuronide, apigenin, diosmetin, coumaric acid, and acacetin). CBDA was employed as a reference standard for the quantification of cannabinoids since its maximum UV adsorption was consistent with that of this class of compounds [11]. The results are illustrated in Figure 2a and expressed as a sum of compounds belonging to the same chemical class in terms of µg of compound in 100 mg of matrix.

The graphs show that the amount of cannabinoids is lower than of the flavonoids content and only traces (around 2 µg/100 mg) of flavonoid aglycones could be detected. Moreover, there was a clear difference between the samples lyophilized and dried in the oven. In particular, the cannabinoids in the freeze-dried samples were about 0.2% of the total content, in agreement with data reported on hemp stems and leaves [9] (ranging from 0.1 to 2%), while for the oven-dried ones the percentage decreased to 0.05%. Regarding the flavonoids, the difference was less marked and ranged between 0.2% and 0.6% for oven- and freeze-drying, respectively, in accordance with the literature [9]. The violin representation (Figure 2b) shows a cluster of freeze-dried samples with a higher content of cannabinoids, which differs from the trend of the other lyophilized matrices. Compared to the previous stages, the samples at the fourth growth stage presented a higher amount of cannabinoids, this being near to the flowering when the synthesis of these phytochemicals increases [20].

### 2.4. Statistical Analysis and Comparison of the Phytochemical Profile of the Different Cannabis sativa Samples

**Figure 3:**
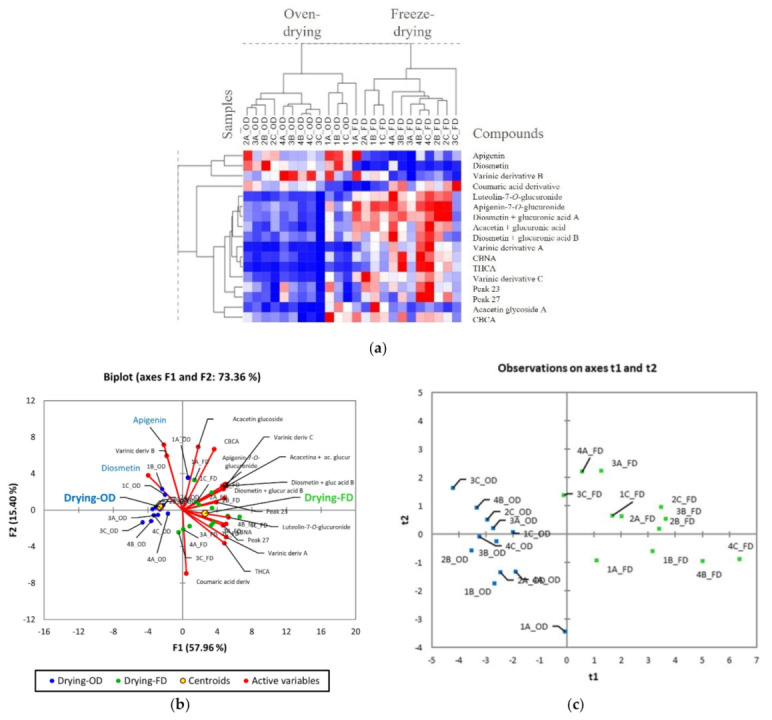
Main results from multivariate statistical analysis on quantification data: (**a**) HC based on Pearson distances and heat-map visualization (24 samples × 17 compounds); (**b**) biplot of the principal component analysis relative to the distribution of the cannabis samples and target compounds according to the methods of drying, growth stages, and land plots (OD and FD samples are highlighted in blue and green, respectively); and (**c**) score plot of partial least squares discriminant according to the method of drying.

The 24 hemp samples under study were characterized by different growth stages, location of growth land plots, and methods of drying (four growth stages × three land plots × two methods of drying). A multivariate statistical analysis was carried out on quantification data (17 target analytes) to investigate the role of these factors as possible discriminants between the samples. First, unsupervised analyses (hierarchical clustering HC and principal component analysis PCA) were performed to investigate the natural groupings existing between the samples, followed by supervised partial least square discriminant analysis (PLS-DA) for targeted statistics. As already mentioned, a clear separation between lyophilized and oven-dried samples is reported in the HC with heatmap visualization (Figure 3a).

The FD samples were characterized by a higher amount of flavonoid glycosides and cannabinoids, which are mainly in the acid form, while the oven-dried samples had a major relative content of the corresponding flavonoids aglycones. As demonstrated by Jimenez-Garcia et al. [37], freeze-drying seems to be a better drying method to preserve flavonoid compounds, especially in the form of glycosides, considering that they may lose the sugar and degrade to the corresponding aglycone if exposed to high temperatures (65 °C) for a prolonged length of time. The same susceptibility is reported for cannabinoids in the acid form, although the degradation normally occurs at a higher temperature than 65 °C. However, the prolonged time of heating in the oven could have affected their content. Moreover, for some cannabinoids the degradation reaction is more complex and it is associated with undetermined side reactions [30]. These data are in agreement with PCA results (Figure 3b), represented in the figure as biplot. The observations (samples) are rationally distributed over the Cartesian plane according to the method of drying (visible along the first principal component F1 from right to left). The biplot show the correlation between the distribution of the samples and the compound content, with a higher amount of flavonoid glycosides and cannabinoids in the freeze-dried samples (green color). To eliminate the strong influence of the drying method on the sample clustering, two different PCA analyses were conducted to study the influence of the growth stage and growth land plot on the phytocomplex of the plant, considered as a data matrix the oven-dried and freeze-dried samples, separately (Figure S5). Interestingly, the growth stage seems to have a lower impact on the contents of the target analytes for both methods of drying. A recent study [20], reported a variation in the content of cannabinoids and flavonoids before and after flowering, this being the rich inflorescences of glandular trichomes, which are devoted to the production of specialized metabolites. As mentioned above, this was confirmed for cannabinoids by the slight increase of their content at the fourth growth stage, while for flavonoids no significant differences were highlighted. As expected, the sample clustering was not affected by the location of the plot, this being in the same geographical area and characterized by similar growing conditions (data not shown). PLS-DA was then applied, focusing on freeze- and oven-drying (Figure 3c), and the variable importance in the projection (VIP) was used to filter compounds having a VIP value ± standard deviation (SD) higher than 1 in the discrimination of each class against the others. Diosmetin, luteolin-7-*O*-glucuronide, varinic derivative A and C, CBNA, diosmetin glucuronide derivative A, and Δ^9^-THCA are reported to be the most informative compounds in the discrimination of oven-dried and freeze-dried samples (see Figure S6).

### 2.5. Evaluation of the Antioxidant Activity of Cannabis sativa Aerial Parts

#### 2.5.1. In Vitro Antioxidant Assays (Scavenging of DPPH^•^ and ABTS^+•^ Radicals)

After the correct characterization and quantification of the main chemicals in the cannabis samples, DPPH^•^ and ABTS^+•^ in vitro colorimetric assays were carried out to investigate the potential antioxidant activity of this matrix. The antioxidant power of flavonoids and phenolic compounds is well-documented, and it is closely linked to their chemical structures, able to reduce free-radical formation and to scavenge free radicals [38]. On the other hand, because of the growing interest in cannabis-derived products, recent studies have been focused on the biological activity of non-psychotomimetic cannabinoids [8,39,40,41], including the antioxidant one [17,42]. As for flavonoids, the antioxidant property is mainly related to the presence of phenolic groups easily oxidized to quinoid forms and of unsaturated bonds found in non-olivetolic fragments of some cannabinoid molecules [43].

**Figure 4:**
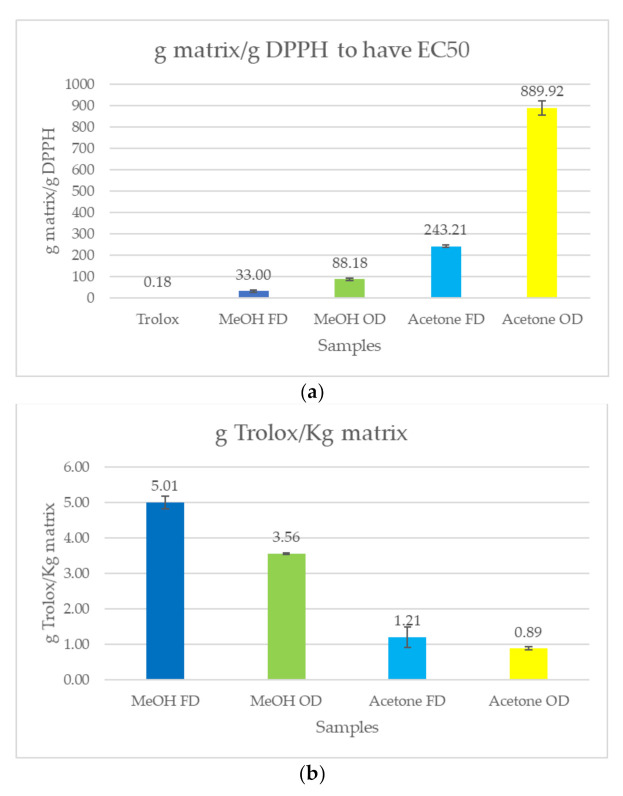
Results of the DPPH^•^ and ABTS^•+^ colorimetric assays: (**a**) g of hemp matrix to obtain 50% of inhibition per 1 g of radical; and (**b**) g equivalent of (±)–6-hydroxy-2,5,7,8-tetramethylchromane-2-carboxylic acid (Trolox) that correspond to 1 kg of hemp matrix.

Since the statistical analysis (Section 2.4) showed that the chemical pattern of the samples is mainly affected by the drying conditions, the colorimetric assays were performed on two different pools of samples, OD and FD, composed by a mixture of the oven-dried and freeze-dried samples, respectively. Moreover, a comparison between the activity of methanolic and acetone extracts was carried out, the first being richer in flavonoids and the second in cannabinoids. Each assay was done in triplicate and the results are summarized in Figure 4.

To build up the DPPH^•^ inhibition curve to extrapolate the EC_50_ (concentration to inhibit 50% of DPPH), a range of concentrations of each extract (OD MeOH, OD acetone, FD MeOH, and FD acetone) was chosen to obtain a linear response (see Figure S7). For the ABTS^+•^ assay, the concentration of the extracts had to fit the Trolox response curve (absorbance as a function of concentration) (Figure S7b). Figure 4a reports the EC_50_ value for DPPH^•^ radicals, and Figure 4b the g equivalent of Trolox for ABTS^+•^. For the DPPH assay, in which Trolox was used as control, the MeOH extract, both the FD and OD, presented a lower value of EC_50_ compared to the acetone one. The higher antioxidant capacity of the methanolic extract may be related to the presence of flavonoids, which are well-known for their antioxidant activity. The antioxidant activity of the samples seemed to also be affected, to a lesser extent, by the sample treatment. In particular, the FD samples, richer in bioactive compounds, exhibited a lower value of EC_50._ Regarding the scavenging of ABTS, a similar behavior was observed, confirming the higher antioxidant power of the FD MeOH extract.

#### 2.5.2. Offline Combination of Antioxidant Assays with HPLC-UV Analysis

To further investigate the contribution of the main components of the extract on the total antioxidant power of the cannabis extracts, an HPLC-UV analysis was coupled to the in vitro colorimetric assays. Methanolic and acetone FD extracts were screened because they have shown to better preserve the chemical composition of the plant. The chromatographic profile was compared before and after the reaction with DPPH^•^ and ABTS^+•^ (see Figure S8) and the % of reduction of the most representative peaks was indicative of the interactions with the radicals and of their scavenging ability. The results are reported in Table 2. The ABTS^+•^ offline analysis was carried out on a different chromatographic column (RP Amide, see Section 3.3) because the peak of ABTS^+•^ radicals coeluted with some of the target analytes. 

Among the flavonoids, luteolin-7-*O*-glucuronide presented the highest interaction with both radicals (peak area reduction >95%), which is probably related to its abundance and to the presence of an additional free hydroxylic group, compared to the other compounds of the same class. According to literature, the ortho position of the two OH groups on the benzene ring facilitates radical delocalization by resonance, justifying the major scavenging activity of this compound [44]. Cannabinoids showed a clear percentage reduction in the acetone extract, where flavonoids were not present, confirming that, although to a lower extent, they contributed to the total antioxidant power of the plant. Literature data report that in some cases cannabinoid acids may present a lower antioxidant activity compared to the neutral form because of the hydrogen-bonding formation between the OH and COOH groups, which may decrease the transport ability of electrons [43]. However, the main cannabinoids found in the extracts were in an acid form and seemed to have a similar antioxidant activity as DPPH^•^ radicals. A higher contribution of varinic derivative A and THCA to the total antioxidant activity was observed in the ABTS^+•^ offline analysis.

### 2.6. Evaluation of the Inhibitory Activity of Cannabis sativa Aerial Parts against Tyrosinase and Elastase Enzymes

**Figure 5:**
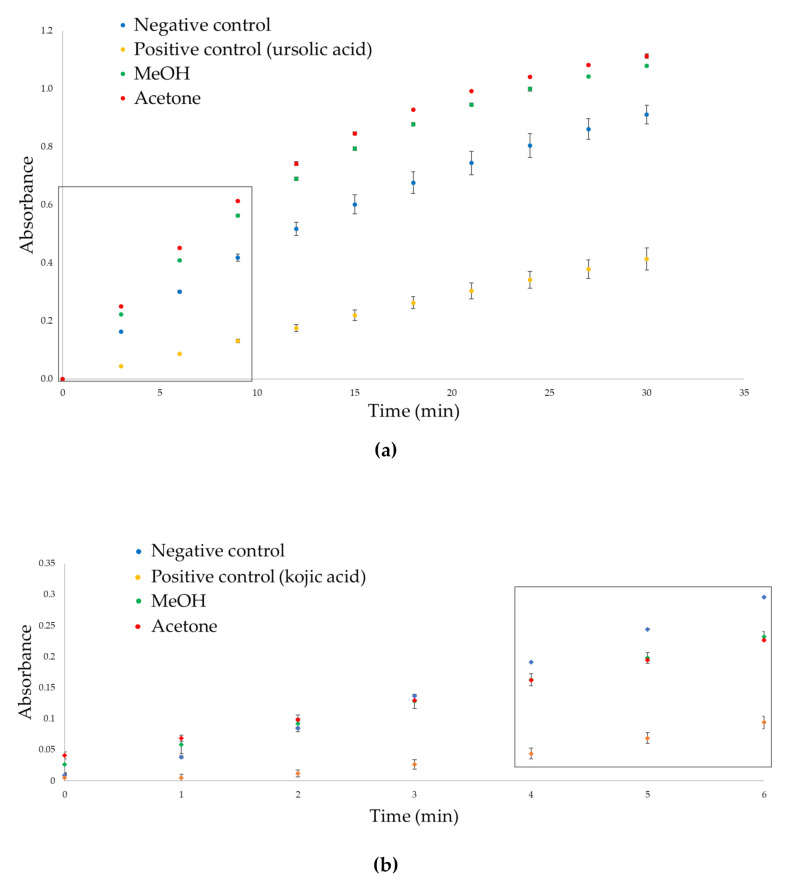
Reaction progress curve for elastase inhibitory activity (**a**) and tyrosinase inhibitory activity (**b**) of negative control, ursolic acid and kojic acid (0.2 mg/mL), MeOH hemp extract, and acetone hemp extract (50 mg/mL). The linearity region is indicated by the black rectangle.

The chemical characterization and the previous in vitro antioxidant assays had demonstrated that the most promising hemp extracts were obtained from FD samples because of the higher content in active compounds. For this reason, preliminary in vitro inhibitory activities against tyrosinase and elastase were tested on the pool composed by the mixture of the FD samples. Mushroom tyrosinase and elastase from porcine pancreas were used for these preliminary screenings of tyrosinase and elastase inhibition activities [45]. Again, the activity of the acetone extract (mainly characterized by non-psychotomimetic cannabinoids) was compared to the methanolic one, which presented a richer profile with both flavonoids and non-psychotomimetic cannabinoids. Figure 5a,b show elastase and tyrosinase reaction progress curves in the absence of any potential inhibitor (i.e., negative control, blue line), in the presence of the investigated extracts (green and red line), and in the presence of ursolic acid and kojic acid, which are elastase and tyrosinase reference inhibitors, respectively (i.e., positive control). 

Both the MeOH and acetone extracts, tested at 50 mg/mL, showed only slight activity against the selected enzymes. In particular, no inhibition was observed of elastase activity. The addition of both extracts to the reaction mix seemed to slightly increase the reaction initial velocities, but further experiments are needed to evaluate the presence of potential elastase activators within the investigated extracts [46]. As regards the tyrosinase inhibitory activity, in agreement with literature data [42,47] both extracts mildly inhibited mushroom tyrosinase activity, suggesting that, although to a minor extent, both flavonoids (higher in the methanolic one) and non-psychotomimetic cannabinoids (higher in the acetone one) could negatively affect tyrosinase activity. Percentages of tyrosinase inhibition of 33.17% ± 2.04% and 38.46% ± 1.36% were observed for the methanol and acetone extracts, respectively, when tested at a concentration of 50 mg/mL. The percentage of enzymatic inhibition was measured as described in Section 3.8.1 after six minutes of incubation to remain under the linearity range of the reaction progress curve, which provides more accurate results [48]. These results were compared to those of kojic acids, which displayed a significantly higher activity (IC_50_ = 0.17 ± 0.015 mg/mL), indicating that the inhibition of the tyrosinase activity of hemp extracts is minimal. 

## 3. Materials and Methods

### 3.1. Plant Material

The dried plant samples (fiber-type *Cannabis sativa* L.) were kindly provided by the Institute of Sciences of Food Production, National Research Council (Grugliasco, Italy). The hemp plants were grown in the Western Po Valley (Italy) and the aerial parts (mainly stems and leaves) were collected at four progressive morphological stages, from mid-vegetative to early flower stage, (see Table S3) on three different land plots (A, B, and C) of 2 m^2^, randomly located in a 2 × 12 m^2^ area. The harvested samples were immediately subjected to oven-drying (in a forced-draft oven at 65 °C to constant weight) and freeze-drying (using a lyophilizer), and subsequently ground into a fine powder to pass a 1 mm screen with a Cyclotec mill (Tecator, Herndon, VA, USA). All the samples were refrigerated at 4 °C to prevent degradation. For the optimization of the extraction method, a mix of all 24 samples was prepared by taking an equal amount from each sample.

### 3.2. Chemical and Reagents

LCMS-grade acetonitrile, HPLC-grade acetonitrile, methanol (MeOH) (>99.9% purity), acetone (>99% purity), formic acid (>98% purity), 1,1-diphenyl-2-picrylhydrazyl radical (DPPH^•^), 2,2′-azinobis (3-ethylbenzothiazoline-6-sulfonic acid) diammonium salt (ABTS), potassium persulphate, Trolox, cannabidiol (CBD), cannabidiolic acid (CBDA), cannabichromenic acid (CBCA), apigenin-7-*O*-glucuronide, and luteolin-7-*O*-glucuronide were supplied by Merck Life Science S.r.l. (Milan, Italy). De-ionized water (18.2 MΩ cm) was obtained from a Milli-Q purification system (Millipore, Bedford, MA, USA). Acacetin, apigenin, diosmetin, and coumaric acid were supplied by Extrasynthese (Genay Cedex, France) and were used as the authentic standard for qualitative and quantitative purposes. Individual standard solutions were prepared by dissolving them in methanol at 1 mg/mL and then diluted at different concentrations to obtain the calibration curves. All these solutions were kept protected from light and refrigerated at 4 °C. For GC-MS analysis, pyridine and BSTFA from Merck Life Science S.r.l. (Milan, Italy) were used for the derivatization of the *Cannabis sativa* L. acetone extracts. For the in vitro colorimetric assays, DPPH^•^ and ABTS^+•^ radicals, kojic acid, sodium phosphate monobasic monohydrate, sodium phosphate dibasic anhydrous, L-tyrosine, mushroom tyrosinase (8503 U/mg), dimethyl sulfoxide (DMSO), trizma base, and N-Succinyl-Ala-Ala-Ala-p-nitroanilide elastase from porcine pancreas (5 U/mg) were purchased from Merck Life Science S.r.l. (Milan, Italy).

### 3.3. Instrumentation and Equipment

A Radwag analytical balance (Radom, Poland) with a minimum readability of 10 mg was used to weight reagents, standards, and samples. An ultrasonic bath (Soltec, Sonica S3 EP 2400), a centrifuge (Remi group, Mumbai, India), a vortex (Thermo Fisher Scientific, Rodano, Italy), a rotavapor (Phoenix instruments, Garbsen, Germany), and a Visiprep™ SPE vacuum manifold (Merck, Milan, Italy) were employed during the extraction procedure. C18 solid-phase extraction (SPE) cartridges (Agilent Technologies, Wilmington, DE, USA) were used for the purification of the extracts from chlorophylls. All the extracts were filtered with filter paper (12 cm in diameter) before SPE, and polyvinylidene fluoride (PVDF) syringe filters from CPS Analitica (Milan, Italy) before injection into the LC system.

A Shimadzu Nexera ×2 UHPLC system was used for qualitative analysis; it was equipped with an SPD-M20A photodiode array detector in series with a Shimadzu LCMS-8040 triple quadrupole system with an electrospray ionization (ESI) source (Shimadzu, Dusseldorf Germany). An Ascentis Express RP-C18 column (15 cm × 2.1 mm, 2.7 μm, Supelco, Bellefonte, PA, USA) was used for quali-quantitative analysis. Mobile phase A was water/formic acid (99.9:0.1, *v*/*v*) and mobile phase B was acetonitrile/formic acid (99.9:0.1 *v*/*v*). The flow rate was 0.4 mL/min and the column temperature was 30 °C. The gradient program was as follows: 0 to 2 min 15% B, 2 to 52 min 15% to 86% B, and 52 to 55 min 86% B. The total analysis time, including pre- and post-running, was 67 min. UV spectra were acquired from 220 to 450 nm. Mass spectrometer operative conditions were as follows: heat block temperature, 200 °C; desolvation line (DL) temperature, 230 °C; nebulizer gas (N_2_) flow rate, 3 L/min; and drying gas (N_2_) flow rate, 15 L/min. Full scan mass spectra were acquired from 50 to 2000 m/z, both in positive and in negative scan modes, with an event time of 0.5 s. When pseudomolecular ions [M + H]^+^ in ESI^+^ or [M − H]^−^ in ESI^−^ were identified, they were subjected to collision (collision energy, −35.0 V for ESI^+^ and 35.0 V for ESI^−^) in product ion scan mode with an event time of 0.2 s. Retention times, UV, and MS spectra were used to identify and tentatively identify the main components of the extracts. These data were compared with those of authentic commercial standards or, when not available, to the literature data (see Table 1).

GC-MS analyses were also carried out for identification purposes using a TRACE GC Ultra gas-chromatograph platform from Thermo equipped with a Thermo 3000 Series Automatic Sampling System (AI 3000) and hyphenated to a Thermo DSQ mass spectrometer with an electron impact source (EI mode). GC-MS analyses were carried out on a capillary MEGA-5 MS column (5% phenyl- and 95% methyl-substituted polysiloxane) with the following characteristics: l: 30 m, *d_c_*: 0.25 mm, and *d_f_*: 0.25 µm (MEGA, Legnano, Italy). GC conditions: injector temperature: 280 °C, injection mode: split, ratio: 1/20; carrier gas: helium, and flow rate: 1 mL/min. Temperature program: from 50 °C to 250 °C at 3 °C min^−1^, and from 250 °C to 300 °C at 10 °C/min. The MS transfer line temperature was set at 270 °C. The single quadruple mass spectrometer was used in full scan mode (mass range: 50–700 amu).

To ensure the absence of any harmful solvents in the final hemp extract, the headspace of the extracts was sampled through solid-phase microextraction (HS-SPME) using a Divinylbenzene/Carboxen/Polydimethylsyloxane (DVB/CAR/PDMS) 50/30 µm film thickness and 2 cm length fiber from Merck (Milan, Italy), and analyzed by GC-MS. Sampling was conducted at room temperature for 15 min. The same GC-MS conditions reported above were used with the exception of the mass range, which was set to 30–300 amu. No traces of residual solvents were found. 

Quantification and DPPH^•^ offline analyses were carried out with a Shimadzu UFLC XR (Shimadzu, Dusseldorf, Germany) equipped with a photodiode array detector SPD-M20A using the same column, mobile phases, flow rate, and gradient program as in the qualitative analysis. For the ABTS^+•^ offline analysis, an Ascentis Express RP Amide column (10 cm × 2.1 mm, 2.7 μm, Supelco, Bellefonte, PA, USA) was employed. The gradient program was as follows: 0 to 3 min 5% B, 3 to 20 min 5% to 15% B, 20 to 30 min 15% to 25% B, 30 to 42 min 25% to 75% B, 42 to 52 min 75% to 100% B, and 52 to 53 min 100% B. The total analysis time including pre- and post-running was 65 min. UV spectra were acquired in the 220–450 nm wavelength range and the resulting chromatograms were integrated at 254 nm to process the analysis for the offline analysis of antioxidant assays and at the λ max of the identified peaks (see Table 1) for quantitative analysis. The calibration curves and the analytical performances of the method are reported in Table S2. All of the analysis was done in triplicate and the analytical performances were measured in terms of repeatability.

All HPLC and GC data were processed using LabSolution software (Shimadzu, Dusseldorf, Germany).

Thermo Spectronic Genesys 6 spectrophotometer (Waltham, MA, USA) was employed for photometric measurement.

### 3.4. Ultrasound-Assisted Solid Liquid Extraction Method

According to the optimized method of extraction (Section 2.1), 5 mL of solvent (MeOH or acetone) were added to 100 mg of dried sample and a US-assisted extraction was performed for 10 min at 40 KHz at room temperature. The liquid phase was then subjected to centrifugation at 4000 rpm for 10 min while the US extraction procedure was repeated twice on the same plant matrix to obtain an exhaustive extraction of the target analytes. After the centrifugation, the supernatant was collected and filtered with filter paper and the solvent was completely evaporated at 40 °C in a rotary evaporator, under vacuum. At this point, the dried extract was subjected to an SPE procedure to eliminate the chlorophylls which can damage the chromatographic column. For this purpose, the extract was reconstituted with 1.5 mL of MeOH/water (40:60, *v*/*v*) and eluted with 8 mL of MeOH/water (85:15, *v*/*v*) through a C18 cartridge (previously activated with 4 mL of MeOH and 4 mL of water). The obtained extract was dried with a gentle nitrogen stream, diluted to 5 mg/mL with MeOH/water (85:15, *v*/*v*), and finally filtered (0.20 μm, PVDF) before the injection in the LC instrument.

### 3.5. Derivatization for GC-MS Analysis 

To prevent the decarboxylation of cannabinoid acids in the injector of the GC, a derivatization of the acetone extract was performed before chromatographic analysis, according to [49]. Also, 80 µL of pyridine and 120 µL of BSTFA were added to 2 mg of the extract and allowed to react for 30 min at 60 °C in a water bath.

### 3.6. Antioxidant Activity 

#### 3.6.1. Scavenging Effect on DPPH^•^ Radicals

The capacity to scavenge the free radical DPPH^•^ was monitored with the method reported by Król [50], with some modifications. A methanolic solution containing the DPPH^•^ radical (25 μg/mL) was prepared to have an absorbance value between 0.65 and 0.72 at 515 nm. The dried extract, obtained with the procedure described in Section 3.4, was diluted in MeOH or acetone and 30 μL of this solution were mixed with 2 mL of the reagent. The mixture was shaken vigorously and left to stand for 30 min at room temperature in the dark (until absorbance values were stable). Reduction of the DPPH^•^ radical was measured by monitoring the absorption decrease at 515 nm. Different concentrations (1, 5, 7.5, and 10 g/L) of the hemp extract were prepared in order to obtain a dose-response curve.

For each concentration, the DPPH scavenging effect was calculated with the following equation:

% scavenging effect = [(A_DPPH_ − A_S_) / A_DPPH_] × 100

where A_DPPH_ is the absorbance of the DPPH^•^ solution and A_S_ is the absorbance of the DPPH^•^ solution after addition of the sample extract. The amount of matrix used to prepare an extract providing 50% inhibition (EC_50_) was calculated by the equation of the line of the dose-response curve. The same procedure was used to obtain the EC_50_ of Trolox, a phenolic compound with a well-known antioxidant capacity, which was used as a positive control to validate the procedure.

#### 3.6.2. Scavenging Effect on ABTS^+•^Radicals

The ABTS method was applied according to Król et al. [50] with some modifications. To form the ABTS^+•^radical, 5 mL of K_2_S_2_O_8_ solution (0.66 mg/mL) was added to 5 mL of ABTS (3.84 mg/mL) and the obtained solution was kept in the dark for 16 h at −20 °C. This solution was then diluted in EtOH/water (50:50 *v*/*v*) to obtain the working reagent with an absorbance of 0.70 ± 0.02 at λ = 734 nm. Once the radical was formed, 2 mL of ABTS^+•^ radical solution was added to 100 μL of the hemp extract (Section 3.4) diluted in EtOH, and the absorbance at λ = 734 nm was measured after 6 min. The ABTS^+•^ scavenging effect was calculated as g equivalent of Trolox per kg of matrix. The Trolox calibration curve was built up by analyzing the standard compound at concentrations ranging from 25 to 100 mg/L, with the same method.

### 3.7. Offline Combination of Antioxidant Assays and HPLC-PDA Analysis

To investigate which compounds of the hemp extract had a higher affinity with DPPH^•^ and ABTS^+•^, an HPLC-UV analysis was carried out before and after the reaction with the radicals. 

#### 3.7.1. DPPH Analysis

To perform DPPH analysis, 30 μL of the hemp extract (diluted in MeOH at the concentration providing the EC_50_) was mixed with 2 mL of DPPH reagent (25 μg/mL), shaken vigorously, and left to stand for 30 min at room temperature in the dark. After the reaction, the solvent was evaporated with a gentle nitrogen steam and the dried extract was diluted with 150 μL of MeOH before injection into the LC system. The chromatographic profile of the extract after reaction with the DPPH^•^ radical solution was compared with that of the same extract before reaction, diluted 1:5.

#### 3.7.2. ABTS Analysis

To perform ABTS analysis, 100 μL of the hemp extract at 2 g/L, diluted in EtOH, was mixed with 2 mL of ABTS working solution, shaken vigorously, and left to stand for 6 min at room temperature in the dark. After the reaction, the solvent was evaporated with a gentle nitrogen steam and the dried extract was diluted with 2000 μL of MeOH before injection into the LC system. The chromatographic profile of the extract after reaction with the ABTS^•^ radical solution was compared with that of the same extract before reaction.

### 3.8. Enzimatic Inhibition Assays

#### 3.8.1. Tyrosinase Inhibition 

The tyrosinase inhibition activity of the FD methanolic and acetone hemp extracts was tested with a colorimetric assay optimized by Zengh et al. [51], with slight modifications. The formation of dopachrome from L-tyrosine was monitored by measuring the absorbance at 475 nm. Both the methanolic and acetone-dried extracts (Section 3.4) were previously dissolved in DMSO to obtain the concentration of 50 mg/mL. Subsequently, 10 μL of this solution was added to 600 μL of phosphate buffer 0.02 M (pH = 6.8) and 400 μL of mushroom tyrosinase 250 U/mL, previously dissolved in a phosphate buffer. The mixture was pre-incubated for 6 min at 30 °C. After the pre-incubation, 500 μL of tyrosine solution in a phosphate buffer (0.1 mg/mL) was added and the absorbance of the mixture was measured. In particular, the absorbance was registered from time “0” to 6 min every minute to obtain a kinetic response curve. A blank solution (without the enzyme) was used to calibrate the instrument. The reaction progress curves were performed for the methanolic and acetone extracts, as well as for the control (10 μL of DMSO instead of the extract) and for a positive control (kojic acid at 0.2 mg/mL) (see Figure 5b).

The initial velocity (v_o_) of each curve, which is equivalent to the slope of the line, was measured and the % of tyrosinase inhibition was determined as follows:

% tyrosinase inhibition = [(slope_control_ − slope_sample_) / slope_control_] × 100

#### 3.8.2. Elastase Inhibition

The elastase inhibition activity of FD methanolic and acetone hemp extracts was tested with a colorimetric assay optimized as reported by Bieth et al. [52], with slight modifications. The elastase inhibition assay is based on the spectrophotometric evaluation of the release of p-nitroaniline from N-succinyl-Ala-Ala-Ala-p-nitroanilide, stimulated by elastase. The formation of p-nitroaniline was determined at 410 nm. The methanolic and acetone-dried extracts (Section 3.4) were dissolved in DMSO to obtain the concentration of 50 mg/mL. Then, 5 μL of this solution were added to 1345 μL of Trizma buffer (pH= 8) and 50 μL of elastase solution 0.6 U/mL (ratio 1:99 in Trizma buffer). During the measurements, the temperature of the elastase solution was kept around 0–4 °C, while the other reagents were at room temperature. The obtained mixture was pre-incubated for 6 min at 30 °C and subsequently 100 μL of substrate 1 mg/mL solution in Trizma buffer was added. The absorbance was registered from time “0” to 30 min every three minutes and a blank solution (without the enzyme) was used to calibrate the instrument. The reaction progress curves were performed for the methanolic and acetone extracts, as well as for the control (5 μL of DMSO instead of the extract) and positive control (ursolic acid, at IC_50_ 5 mg/mL) (see Figure 5a). The initial velocity (v_o_) of each curve, which is equivalent to the slope of the line, was measured and the % of elastase inhibition was determined with the same equation reported for tyrosinase (Section 3.8.1).

### 3.9. Statistical Analysis

Experimental designs and the elaboration of the violin plots were carried out by using Statgraphics^®^ 18–X64 software. Statistical analysis and chemometrics were obtained with XLSTAT statistical and data analysis solution (Addinsoft 2020, New York, NY, USA). while the heatmap was created by using Morpheus software (https://software.broadinstitute.org/morpheus (accessed on 7 January 2022)). Excel software (Microsoft Office, v.2016) was employed for the remaining calculations.

## 4. Conclusions

The non-volatile fractions of fiber-type *Cannabis sativa* L. aerial parts (collected before flowering) were carefully characterized by UHPLC-UV-ESI-MS/MS using an optimized method of extraction. The leaves and stems of the plant proved to be a valuable source of specialized metabolites (chiefly flavonoids and non-psychotomimentic cannabinoids) and their antioxidant activity was evaluated by colorimetric in vitro assays (DPPH^•^ and ABTS^+•^). Flavonoids presented the major influence on the antioxidant activity, but cannabinoids also showed a contribution to the total antioxidant power of the hemp extracts. The in vitro inhibitory activity against tyrosinase and elastase was also tested on methanolic and acetone extracts. No inhibition was perceived against elastase activity and a slight inhibition of the tyrosinase enzyme was observed for both of the extracts at high concentrations. The method of drying, fundamental for storing the plant material, plays a fundamental role in preserving the chemical composition of the plant, especially for thermolabile compounds. The variation in the chemical patterns among the investigated growth stages is less marked and only the content of cannabinoids presented a slight increase at the stage closer to flowering. These results suggested that fiber-type *Cannabis sativa* L. aerial parts can be considered a valuable natural ingredient, considering that their chemical composition seems to be independent of the growth stage of harvesting, provided that they are collected before the flowering stage. In this sense, further studies on the biological activity of these extracts are necessary to confirm and support the preliminary results obtained with in vitro assays and to evaluate potential toxic effects at the tested concentrations. In particular, the isolation of enriched fractions of the more active compounds and further tests on cellular and animal models may provide a significant contribution for safe and future applications of hemp extracts.

## Figures and Tables

**Table 1:** List of identified and putatively identified compounds in fiber-type *Cannabis sativa* L. aerial parts. For each analyte, retention time, UV maximum, pseudomolecular ions, and fragment ions were obtained by product ion scan mode (PIS) and identified or tentatively identified compound names are given. Identification confidence values and references are also included. The compounds confirmed with the injection of authentic commercial reference standards and in bold.

N°	t^r^ (min)	λ max (nm)	[M + H]^+^ *m*/*z*	[M − H]^−^ *m*/*z*	Mol. Weight (g/mol)	M^2+^ *m*/*z*	M^2−^ *m*/*z*	Aglycon (g/mol)	Compound Name	Identification Levels ^§^	Ref.
1	5.0	346/252	463	461	462	287	285	286	**Luteolin-7-*O*-glucuronide**	1	[4,9,13]
2	7.2	336/266	447	445	446	271	269	270	**Apigenin-7-*O*-glucuronide**	1	[4,9,13]
3	7.5	345/250	477	475	476	301	299	300	Diosmetin glucuronide derivative A	2	[4,9,13]
4	8.1	345/250	477	475	476	301	299	300	Diosmetin glucuronide derivative B	2	[4,9,13]
5	9.4	335/267	623	621	622	285	283	284	Acacetin glycoside A	2	[4,9,13]
6	10.8	332/267	593	591	592	285	283	284	Acacetin glycoside B	2	[4,9,13]
7	11.0	328/267	607	605	606	285	283	284	Acacetin glycoside C	2	[4,9,13]
8	12.1	334/267	461	459	460	285	283	284	Acacetin glucuronide derivative	2	[4,9,13]
9	13.2	336/266	271	269	270	/	/	/	**Apigenin**	1	[4,9,13]
10	14.3	343/252	301	299	300	/	/	/	**Diosmetin**	1	[4,9,13]
11	17.7	268/279	/	/	/	/	/	/	Not identified	/	/
12	18.2	268/279	/	/	/	/	/	/	Not identified	/	/
13	18.4	268/279	/	/	/	/	/	/	Not identified	/	/
14	20.1	267/334	285	283	284	/	/	/	**Acacetin**	1	[4,9,13]
15	23.9	310	/	667	/	/	163	/	Coumaric acid derivative	2	[4]
16	36.4	341/274	437	435	436	313	/	/	**Cannflavin A**	1	[12,13,33]
17	36.6	220/269/306	359	357	358	/	/	/	**Cannabidiolic acid (CBDA)**	1	[12,33]
18	37.9	220/268/305	361	359	360	219	/	/	Cannabigerolic acid (CBGA)	2	[12,33]
19	38.1	263/328	327	325	326	191	/	/	Cannabinoid	3	[12,33]
20	40.2	220/271/305	331	329	330	191, 313, 257, 233	/	/	Varinic derivative A	2	[12,33]
21	41.8	220/272/304	331	329	330	191, 257, 233	/	/	Varinic derivative B	2	[12,33]
22	42.1	220/271/305	331	329	330	191, 257, 233	/	/	Varinic derivative C	2	[12,33]
23	43.8	268/306	347	345	346	205, 175	/		Cannabinoid	3	[12,33]
24	44.0	/	355	353	354	337, 281	/	/	Cannabinolic acid (CBNA)	2	[12,33]
25	46.1	220/271/304	359	357	358	219, 243	/	/	Δ^9^-Tetrahydrocannabinolic acid (Δ^9^-THCA)	2	[12,33]
26	47.9	/	359	357	358	219, 341, 261	/	/	**Cannabichromenic acid (CBCA)**	1	[12,33]
27	48.5	266/305	375	373	374	233	/	/	Cannabinoid	3	[12,33]

^§^ Identification confidence according to the request of the Chemical Analysis Working Group (CAWG, 2007) [34]: Level 1, identified compound (a minimum of two independent and orthogonal data, such as retention time and mass spectrum) compared directly relative to an authentic commercial reference standard; Level 2, putatively annotated compound (compound identified by analysis of spectral data and/or similarity to data in a public database); and Level 3, putatively characterized compound class level.

**Table 2:** Percentage of peak area reduction (±SD), after the reaction with DPPH^•^ and ABTS^+•^ radicals, for MeOH and acetone extracts.

Compounds	% Peak Reduction			
	DPPH		ABTS	
	MeOH	Acetone	MeOH	Acetone
Luteolin-7-*O*-glucuronide	>95(±0.09)	/	>95(±0.05)	/
Apigenin-7-*O*-glucuronide	19.6(±2.73)	/	18.3(±1.13)	/
Diosmetin glycoside A	24.7(±2.31)	/	37.4(±2.24)	/
Diosmetin glycoside B	22.3(±0.15)	/	/	/
Acacetin glycoside A	25.7(±0.54)	/	89.2(±3.04)	/
Varinic derivative A	27.9(±1.44)	40(±5.65)	/	76.5(±5.89)
Varinic derivative C	19.2(±1.38)	31(±2.28)	/	36(±4.94)
THCA	22.5(±3.77)	42.4(±1.11)	/	72.7(±5.73)
CBCA	42.1(±0.09)	52.1(±0.21)	/	35.3(±6.11)

## Data Availability

The data presented in this study are available on request from the corresponding authors. The data are not publicly available because their elaboration are all reported in the manuscript.

## Supplementary Materials

The following are available online at https://www.mdpi.com/article/10.3390/plants11030419/s1: Table S1: Variables considered in the experimental design (2^n^ screening study), Table S2: Quality analytical parameters of the HPLC-UV method, Table S3: Classification of the hemp samples analyzed in this study: acronymous, growth stage, land plot, and drying method, Table S4: Parameters of the in vitro tyrosinase inhibitory assay, Figure S1: Chromatographic profile at 254 nm of methanolic and ethanolic extraction of fiber-type *Cannabis sativa* L. aerial parts at 5 mg/mL, Figure S2: Pareto charts obtained in the screening of the main variables for the methanolic (**a**) and acetone extraction (**b**), Figure S3: Representative MS spectrum of peak 21 in ESI^+^ (**a**) and PIS fragmentation (**b**) of pseudomolecular ion 331m/z (putatively identified as acid varinic derivative according to [18]; Figure S4: GC-MS profile of the FD acetone hemp extract, after derivatization with BSTFA (**a**) and MS spectra of selected peak in the hemp extract (**b**), Figure S5: PCA elaboration of oven-dried (**a**) and freeze-dried (**b**) hemp samples, according to the growth stages, Figure S6: Variables’ importance in the projection (VIP) for the discrimination of oven-dried and freeze-dried samples; Figure S7: Representative response curve of FD MeOH extract (**a**) and Trolox (**b**) used to measure the scavenging effect on DPPH^•^ and ABTS^+•^radicals; and Figure S8: Representative chromatogram of FD MeOH hemp profile before and after the reaction with DPPH^•^ radicals (**a**) and FD acetone hemp profile before and after the reaction with ABTS^+•^ (**b**).
