# Supplementary material for: Characterization and Biological Activity of Fiber-Type Cannabis sativa L. Aerial Parts at Different Growth Stages
Source: Plants (Basel). 2022 Feb 3;11(3):419. doi: 10.3390/plants11030419 (PMC8838183; doi:10.3390/plants11030419)
Supplement: Supplementary file 1 [file plants-11-00419-s001.zip › plants-1576975-supplementary.pdf]

# Supplementary materials

# Characterization and biological activity of fiber-type *Cannabis sativa* L. aerial parts at different growth stages

**Giulia Mastellone [1,†], Arianna Marengo [1,†], Barbara Sgorbini [1], Federica Scaglia [1], Francesca Capetti [1], Francesco Gai [2], Pier Giorgio Peiretti [2], Patrizia Rubiolo [1], Cecilia Cagliero [1,*]**

[1] Department of Drug Science and Technology, University of Turin, I-10125, Turin, Italy; giulia.mastellone@unito.it, arianna.marengo@unito.it, barbara.sgorbini@unito.it, federica.scaglia@edu.unito.it, francesca.capetti@unito.it, patrizia.rubiolo@unito.it

[2] Institute of Sciences of Food Production, National Research Council, 10095, Grugliasco, Italy; francesco.gai@ispa.cnr.it, piergiorgio.peiretti@ispa.cnr.it

† Both authors contributed equally to this work

* Correspondence: cecilia.cagliero@unito.it; Tel.: +39 011 6707133

**Table S1.** Variables considered in the experimental design ($2^n$ screening study).

| Variable | Low value | High value |
|---|---|---|
| Sample amount (mg) | 100 | 500 |
| Volume of solvent (mL) | 5 | 50 |
| Water in the solvent (%) | 0 | 50 |
| Extraction time with US (min) | 10 | 30 |

**Table S2.** Quality analytical parameters of the HPLC-UV method.

| Compound | λ (nm) | Linear range (mg/L) | $R^2$ | Calibration curve equation | Peak quantified |
|---|---|---|---|---|---|
| Luteolin-7-*O*-glucuronide | 346/252 | 25-150 | 0.9989 | y = 14487x - 24394 | 1 |
| Apigenin-7-*O*-glucuronide | 336/266 | 10-350 | 0.9986 | y = 20488x + 47085 | 2 |
| Apigenin | 336/266 | 0.1-25 | 0.9989 | y = 35166x – 2365.2 | 9 |
| Diosmetin | 343/252 | 0.1-25 | 0.9970 | y = 37180x - 1940.9 | 3, 4, 10 |
| Acacetin | 267/334 | 1-25 | 0.9990 | y = 32388x + 9461.5 | 5, 8 |
| Coumaric acid | 310 | 1-10 | 0.9950 | y = 50388x + 22980 | 15 |
| CBDA | 220/270/310 | 5-50 | 0.9999 | y = 13839x - 21.947 | 20, 21, 22, 23, 24, 25, 26, 27 |

**Table S3.** Classification of the hemp samples analysed in this study: acronym, growth stage, land plot and drying method.

| Sample | Growth stage | Land plot[a] | Drying method |
|---|---|---|---|
| 1A_OD | Mid vegetative | A | Oven-drying[b] |
| 2A_OD | Late vegetative | A | Oven-drying |
| 3A_OD | Shooting | A | Oven-drying |
| 4A_OD | Early flower | A | Oven-drying |
| 1B_OD | Mid vegetative | B | Oven-drying |
| 2B_OD | Late vegetative | B | Oven-drying |
| 3B_OD | Shooting | B | Oven-drying |
| 4B_OD | Early flower | B | Oven-drying |
| 1C_OD | Mid vegetative | C | Oven-drying |
| 2C_OD | Late vegetative | C | Oven-drying |
| 3C_OD | Shooting | C | Oven-drying |
| 4C_OD | Early flower | C | Oven-drying |
| 1A_FD | Mid vegetative | A | Freeze-drying[c] |
| 2A_FD | Late vegetative | A | Freeze-drying |
| 3A_FD | Shooting | A | Freeze-drying |
| 4A_FD | Early flower | A | Freeze-drying |
| 1B_FD | Mid vegetative | B | Freeze-drying |
| 2B_FD | Late vegetative | B | Freeze-drying |
| 3B_FD | Shooting | B | Freeze-drying |
| 4B_FD | Early flower | B | Freeze-drying |
| 1C_FD | Mid vegetative | C | Freeze-drying |
| 2C_FD | Late vegetative | C | Freeze-drying |
| 3C_FD | Shooting | C | Freeze-drying |
| 4C_FD | Early flower | C | Freeze-drying |

[a] 2 $m^2$ subplots randomly located in 2×12 $m^2$ plot

[b] Forced-draft oven to constant weight at 65°C

[c] Lyophilizer

**Table S4.** Parameters of the *in-vitro* tyrosinase inhibitory assay.

| Sample | Slope | $R^2$ | Calibration curve equation |
|---|---|---|---|
| Negative control (no extract) | 0.052 | 0.999 | y = 0.052x - 0.0172 |
| Positive control (kojic acid) | 0.025 | 0.999 | y = 0.0255x - 0.0522 |
| MeOH hemp extract | 0.035 | 1 | y = 0.0348x + 0.0241 |
| Acetone hemp extract | 0.032 | 1 | y = 0.032x + 0.0345 |

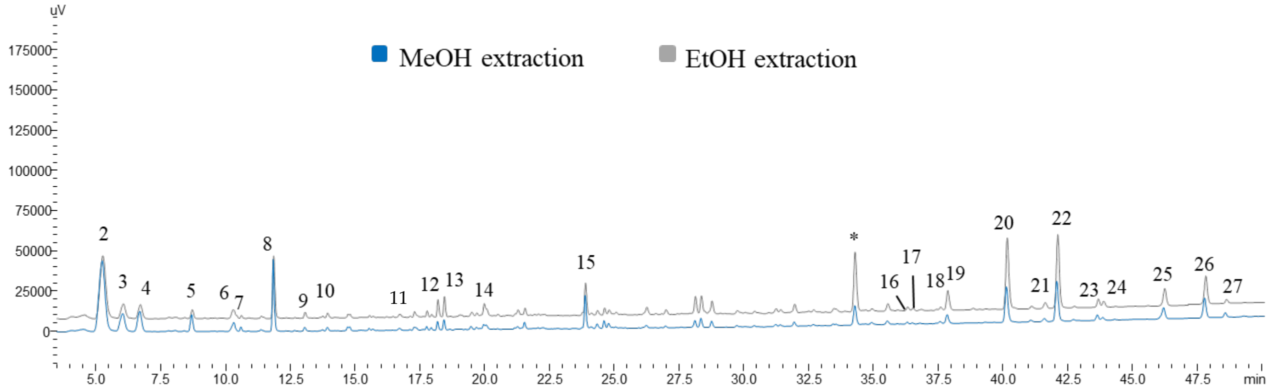


**Figure S1.** Chromatographic profile at 254 nm of methanolic and ethanolic extraction of fiber-type *Cannabis sativa* L. aerial parts at 5 mg/mL. For peaks identification, see Table 1.

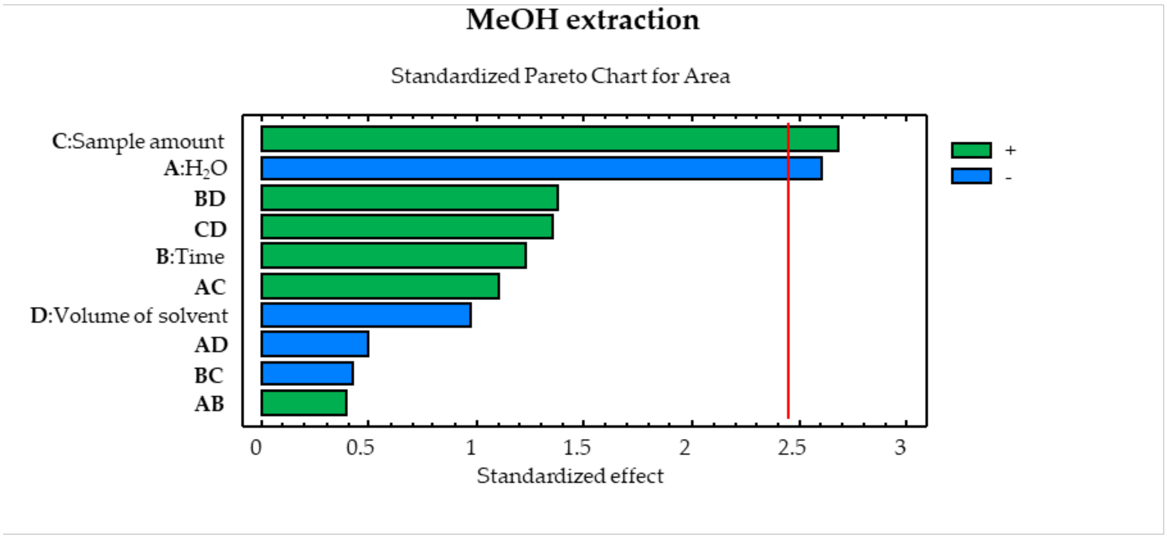


(**a**)

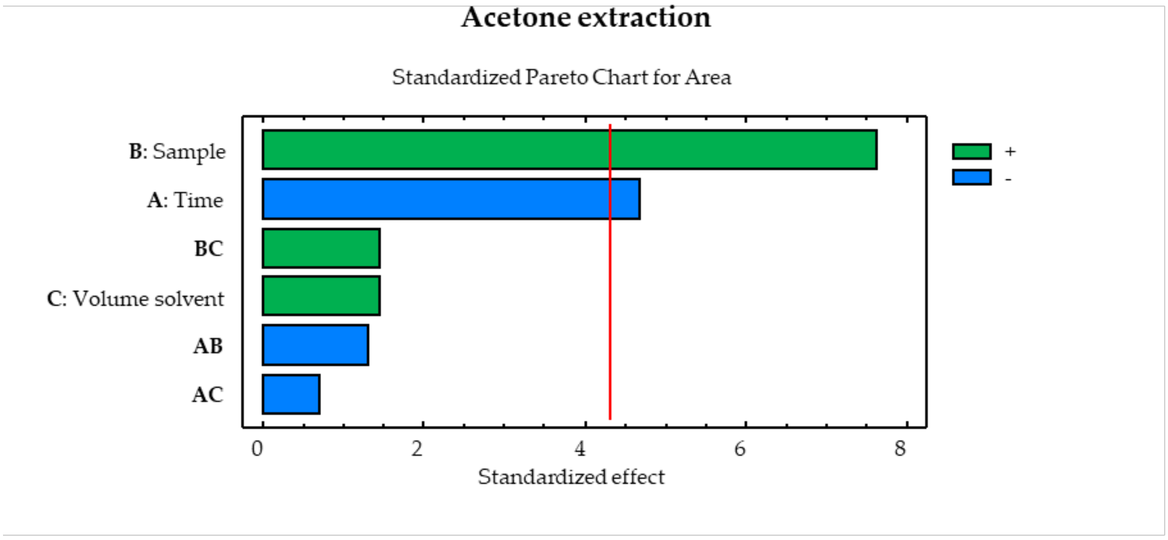


(**b**)

**Figure S2.** Pareto charts obtained in the screening of the main variables for the methanolic (a) and acetone extraction (b).

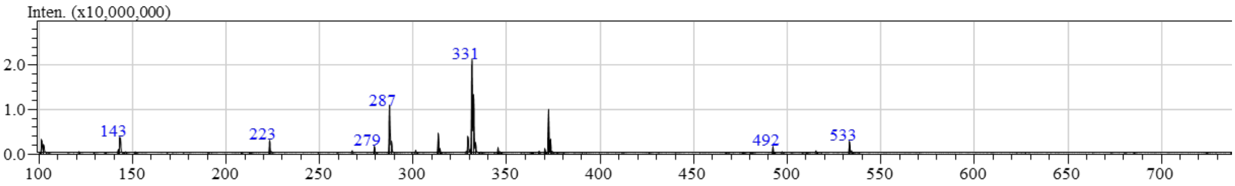


(**a**)

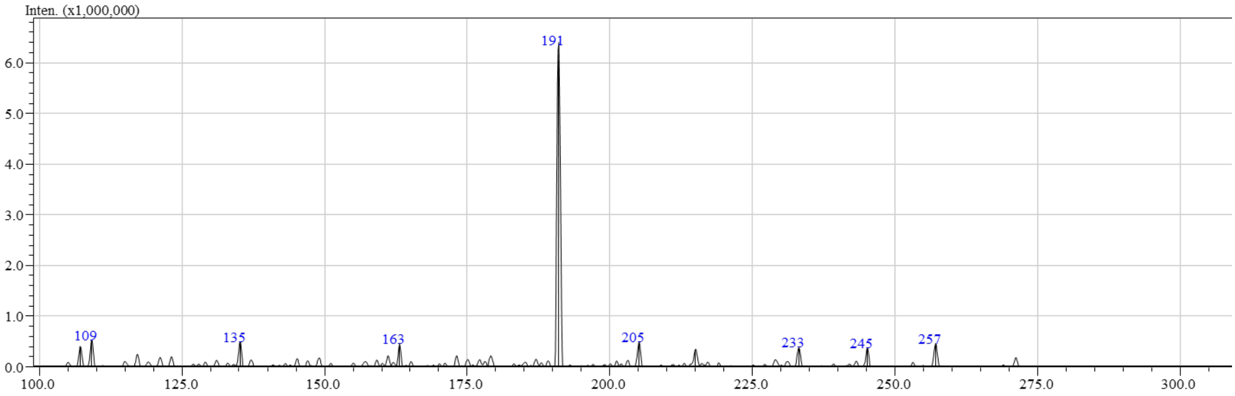


(**b**)

**Figure S3.** Representative MS spectrum of peak 21 in ESI+ (a) and PIS fragmentation (b) of pseudomolecular ion 331m/z (putatively identified as varinic acid derivative according to [32]).

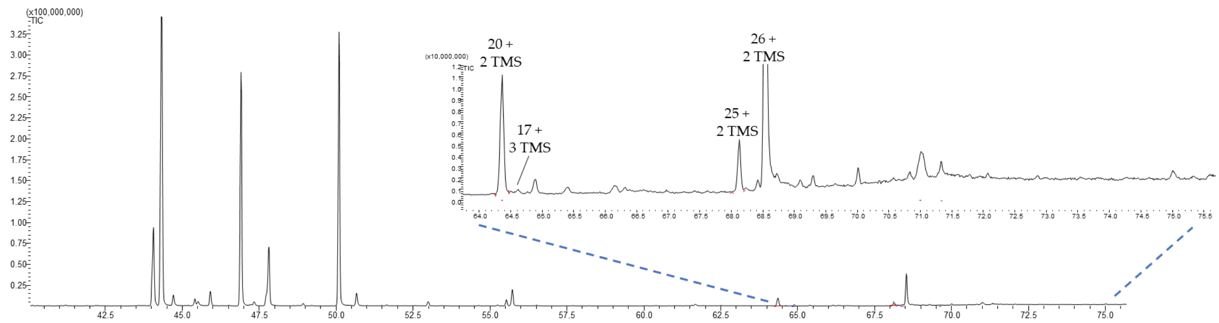


(a)

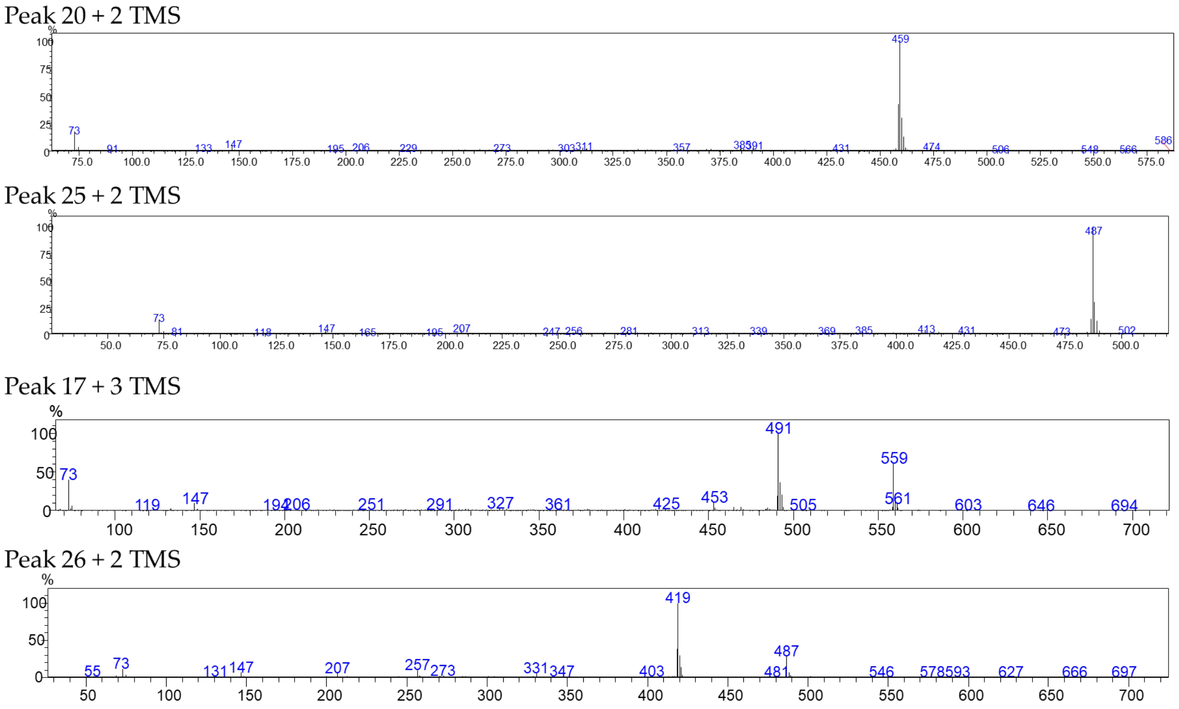


(**b**)

**Figure S4.** GC-MS profile of the FD acetone hemp extract, after derivatization with BSTFA (a) and MS spectra of selected peak in the hemp extract (b). TMS (trimethylsilyl). See table 1 for peak numbers.

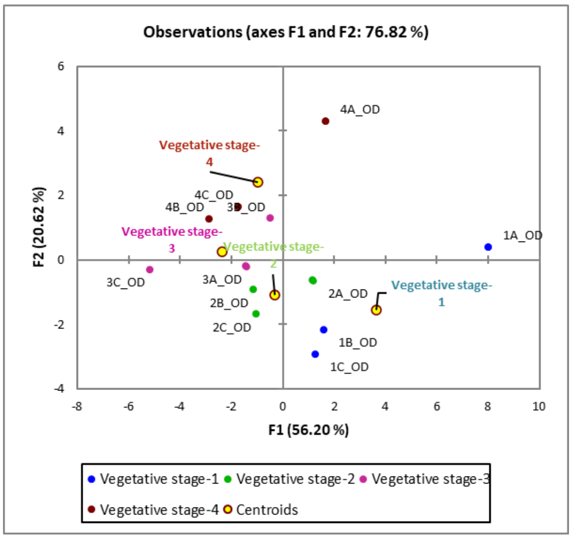


(a)

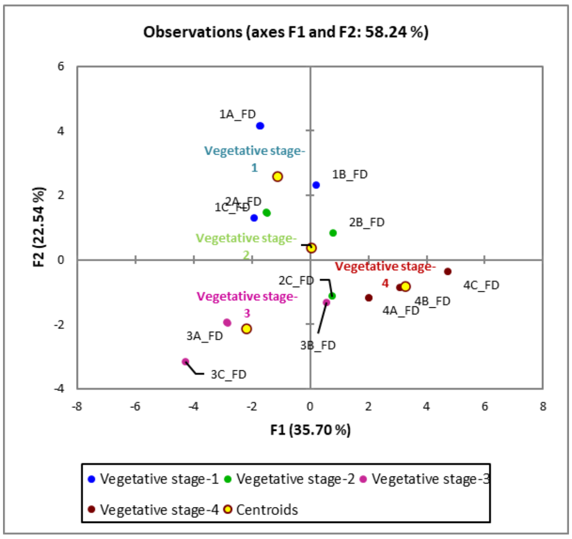


(b)

**Figure S5.** PCA elaboration of oven-dried (a) and freeze-dried (b) hemp samples, according to the growth stages.

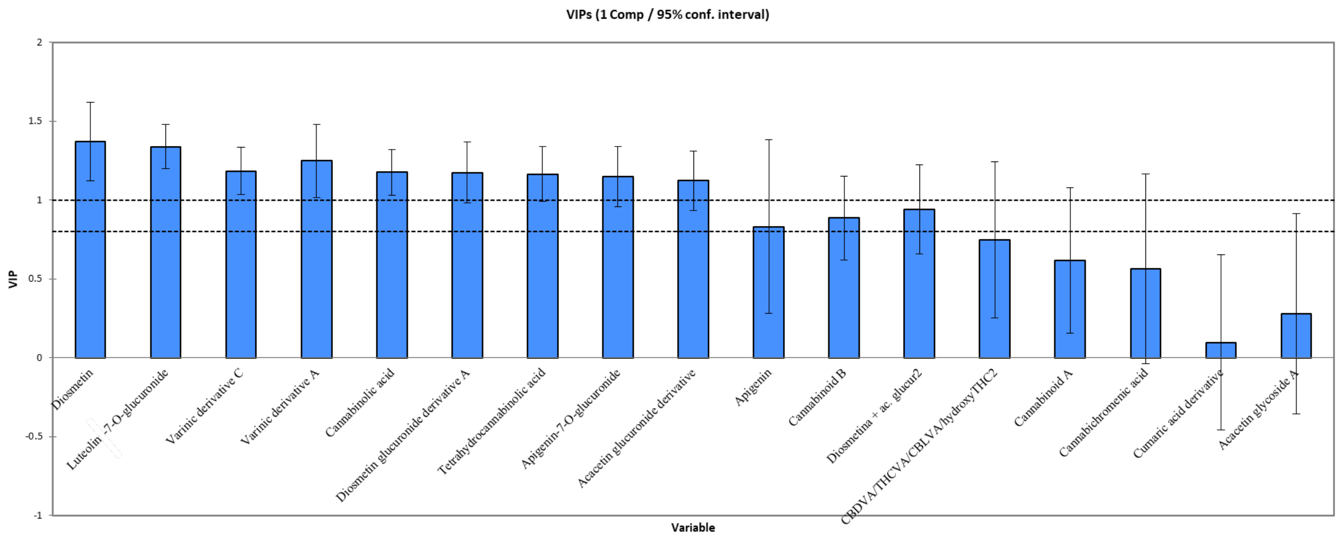


**Figure S6.** Variables importance in the projection (VIP) for the discrimination of oven-dried and freeze-dried samples.

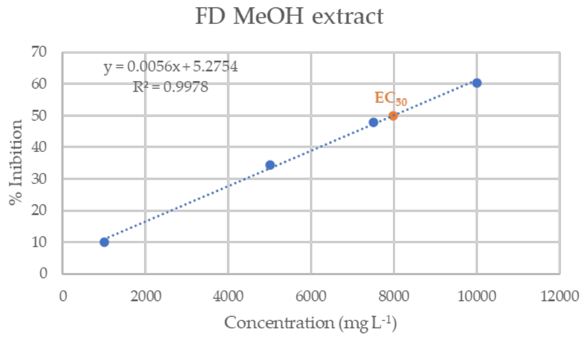


(a)

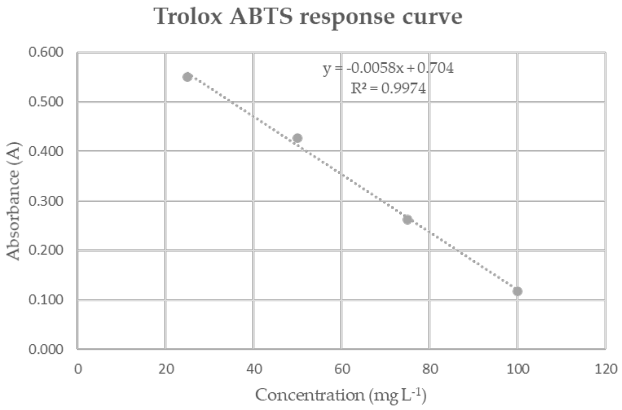


(**b**)

**Figure S7.** Representative response curve of FD MeOH extract (a) and Trolox (b), used to measure the scavenging effect on DPPH$^{\bullet}$ and ABTS$^{+\bullet}$radicals, respectively.

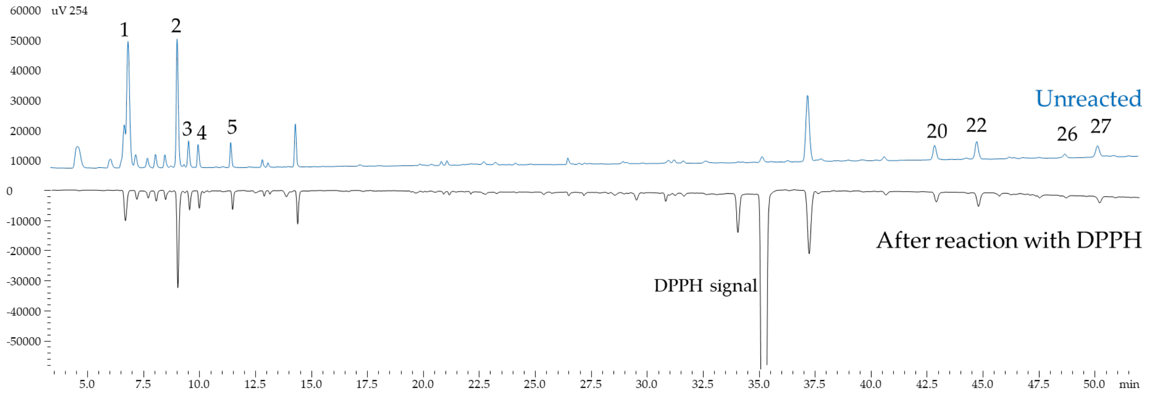


**(a)**

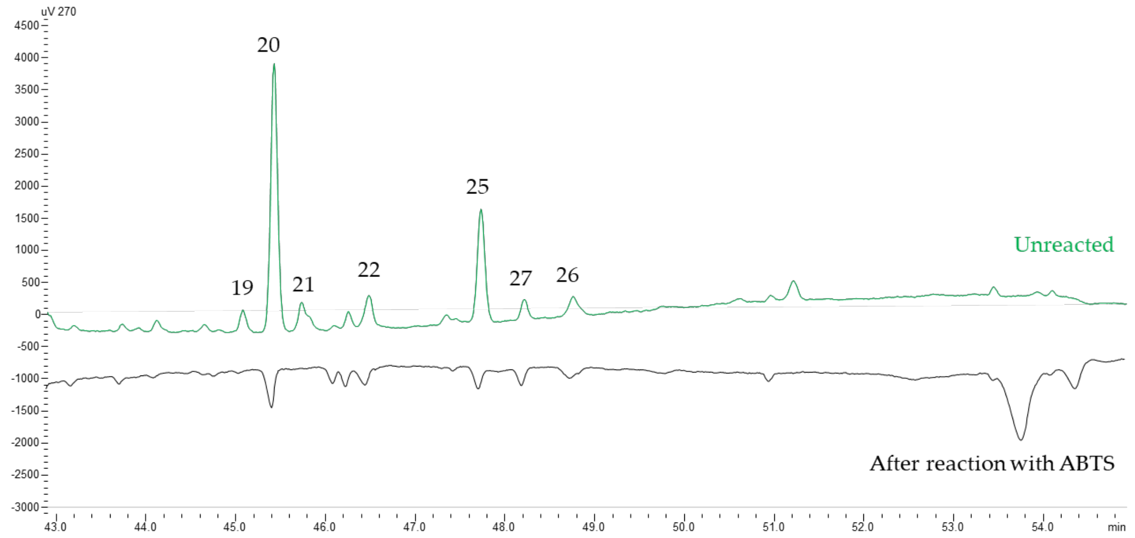


**(b)**

**Figure S8.** Representative chromatogram of FD MeOH hemp profile before and after the reaction with DPPH$^{\bullet}$ radicals on C18 column (a) and FD acetone hemp profile before and after the reaction with ABTS$^{+\bullet}$ on RP-Amide column (b).
